# Major mental disorders among family caregivers: a nation-wide register-based study

**DOI:** 10.1080/02813432.2026.2671956

**Published:** 2026-05-17

**Authors:** Meri-Tuuli Lehmuskallio, Merja K. Laine, Hannu Kautiainen, Hannu Koponen, Tuija M. Mikkola

**Affiliations:** ^a^ Department of General Practice and Primary Health Care, University of Helsinki and Helsinki University Hospital, Helsinki, Finland; ^b^ Folkhälsan Research Center, Helsinki, Finland; ^c^ Primary Health Care Unit, Kuopio University Hospital, Kuopio, Finland; ^d^ Department of Psychiatry, University of Helsinki and Helsinki University Hospital, Helsinki, Finland; ^e^ Clinicum, Faculty of Medicine, University of Helsinki, Helsinki, Finland

**Keywords:** Aging, family caregiver, informal caregiver, mental disorder, psychiatric diseases, register-based study, psychiatric morbidity

## Abstract

**Purpose:**

Evidence of (informal) family caregivers’ probability for major mental disorders remains limited. This study aimed to examine the association between high-intensity informal caregiving and major mental disorders using register-based data.

**Methods:**

This study included all recipients of the family caregiver’s allowance in Finland in 2012 (*n* = 42,256) and matched controls (*n* = 83,618). Information on diagnoses of mental disorders treated in hospitals or specialized healthcare settings was obtained from the National Care Register for the period 2012–2017.

**Results:**

Overall, 5036 (11.9%) caregivers received at least one diagnosis of a major mental disorder. Caregivers exhibited a lower likelihood of major mental disorders compared to controls; however, from youth to age 70, caregivers were more likely to experience a major mental disorder compared with controls. Caregivers had a higher incidence rate ratio (IRR) for major psychiatric conditions, particularly depressive and mood disorders (IRR 1.33, 95% confidence interval [95% CI] 1.26–1.41) and anxiety-related disorders (IRR 1.37, 95% CI 1.29–1.46). Male caregivers had a higher IRR of mental and behavioral disorders related to psychoactive substance use compared with female caregivers (IRR 3.04, 95% CI 2.66–3.47). By contrast, male caregivers had a lower IRR of depressive and mood disorders than female caregivers (IRR 0.74, 95% CI 0.66–0.82).

**Conclusions:**

This study enhances our understanding of family caregivers’ likelihood for specific mental disorders. Improved knowledge of these probabilities may facilitate the development of proactive strategies to protect caregivers’ mental health and coping capacity before clinically significant problems emerge.

## Introduction

Demographic trends, such as an aging population; extended lifespans with chronic conditions and disabilities; and underfunded, fragmented health and social care systems, have increasingly shifted the caregiving burden onto family members [1]. Family caregivers are individuals who, despite lacking formal training or professional experience, provide care or support to a spouse, child, sibling or partner who is unable to care for themselves due to illness, disability or aging [1,2]. The terms ‘informal caregiver’ and ‘family caregiver’ are often used interchangeably; in this study, we use ‘caregiver’ to refer both.

While caregiving can bring rewards, such as personal satisfaction, increased self-efficacy and a sense of purpose, it can also be burdensome and negatively affect caregivers’ health and wellbeing [1,3].

Previous survey-based studies have shown that caregivers are at a higher risk than non-caregivers for stress-related symptoms and common mental disorders, particularly depressive and anxiety disorders [4–6]. However, existing evidence relies primarily on survey data, often focusing on specific age groups or diseases, or assesses self-reported symptoms rather than diagnosed mental disorders [7–10]. These limitations highlight the need for register-based studies that include comprehensive diagnostic information on caregivers [7–10].

Previous research indicates that higher-intensity informal caregiving is associated with poorer wellbeing and worse physical and mental health outcomes among caregivers. By contrast, low- or moderate-intensity caregiving does not appear to be associated with similar adverse effects [5,11–15].

Some studies also suggest that the adverse mental health effects of caregiving may differ by gender. More specifically, female caregivers consistently exhibit higher levels of psychological distress and a higher prevalence of depressive symptoms than non-caregivers, while comparable effects have not been observed among men [16–19].

To the best of our knowledge, no register-based studies using diagnostic codes have examined the probability of major mental disorders among caregivers. In previous studies, we scrutinized the relationship between family caregiving, mortality, work incapacity and the use of psychoactive medications [20–23]. In this study, we aimed to explore the relationship between high-intensity family caregiving and major mental disorders using register-based data. We further assessed whether this association varies by age, sex and diagnostic category.

## Materials and methods

### Context

In Finland, the number of family caregivers has shown consistent annual growth [24]. An estimated 1.2 million adults, more than a quarter of the population aged 20 years and older, provide support to a close person in need outside the formal service system [25]. of these, over 51,000 individuals receive a caregiver’s allowance and provide high-intensity informal care [24,26].

The granting of a family caregiver’s allowance is regulated by Finnish legislation. In Finland, Wellbeing Services Counties (prior to 2023, ‘municipalities’) may grant this allowance to individuals who provide care or assistance at home due to the care recipient’s functional limitations, illness, disability or similar reasons. The eligibility criteria are determined by the Wellbeing Services County. Eligibility is assessed based on how demanding and binding the caregiving tasks are, as well as the caregiver’s health and functional capacity. These assessments are typically conducted by a social worker during a home visit. The caregiver’s income or employment status does not affect eligibility.

A report from the Finnish Ministry of Social Affairs and Health, states that 80% of caregivers engage in nearly continuous, demanding caregiving tasks [27]. A Finnish survey further indicated that 85% of formally recognized caregivers provided care for at least seven hours per day [28]. During 2023 about half of the care recipients were aged 75 years or older, and slightly less than one-fifth were under 18 [24]. Caregivers are predominantly spouses, followed by parents and adult children of the care recipient [29].

### Materials

We identified all officially recognized caregivers in Finland based on their receipt of a family caregiver’s allowance in 2012, as recorded in the Finnish Tax Administration register. A total of 42,256 caregivers were identified, including 29,846 females with a mean age of 66 years and 12,410 males with a mean age of 71 years. Two controls per caregiver were selected without replacement from the National Population Register (maintained by the Digital and Population Data Services Agency), matched by birth year, sex and municipality of residence. After excluding individuals in institutional care, the final control group consisted of 83,618 individuals (see Mikkola et al. [21] for details). Data linkages were performed by registry-keeping authorities using personal identification codes. Before delivering data to the research group, all data were pseudonymized, and personal identification codes were removed.

Data on birth year were obtained from the Population Register Center. Educational attainment was determined by the highest degree obtained based on Statistics Finland data by the year 2012. Information on annual earned income, caregiver’s allowance and capital income in 2012 was retrieved from the Finnish Tax Administration’s register. For the descriptive statistical analysis, employment status was inferred from socioeconomic status (SES) data from Statistics Finland and from income records. Socioeconomic position was evaluated based on an individual’s life situation (such as economic activity or pensioner status), occupation and occupational status in 2012 [30]. This variable was then reclassified into three employment status groups: (1) employed/student, (2) unemployed/part-time employment and (3) pensioner. SES variable was calculated as the average of rank-normalized years of education and total income [21]. We used the Van der Waerden rank-based normalization to yield standardized scores for each of the two variables (years of education and income) [31] and then computed the average of those scores. Data on caregiving status and background variables are from baseline.

Primary and secondary psychiatric diagnoses were obtained from the Care Register for Health Care, maintained by the Finnish Institute for Health and Welfare, covering the period 2012–2017. The register includes information on patients in inpatient care at hospitals and health centers, as well as those discharged from inpatient care, day surgeries and specialized outpatient care providers.

Diagnoses were coded according to the International Statistical Classification of Diseases and Related Health Problems, tenth revision (ICD-10). Here, we focused on diagnoses within group F: mental, behavioral and neurodevelopmental disorders. While the total number of group F diagnoses included all diagnoses in this category, we specifically classified and analyzed the following subgroups: mental disorders caused by known physiological conditions, such as dementia (F00–F09); mental and behavioral disorders due to psychoactive substance use (F10–F19); schizophrenia, schizotypal, delusional and other non-mood psychotic disorders (F20–F29) (hereafter non-affective psychotic disorders); manic episodes and bipolar disorder (F30–F31); depressive episodes, major depressive disorders, recurrent, persistent mood (affective) disorders and unspecified mood (affective) disorders (F32–F39) (hereafter depressive and mood disorders); anxiety, dissociative, stress-related and somatoform mental disorders (F40–F45) (hereafter anxiety-related disorders); eating disorders (F50); specific (adult) personality disorders (F60–F61) and hyperkinetic disorders (F90), with attention-deficit hyperactivity disorder (ADHD) presenting the most common subcategory.

These subgroups were selected to provide a comprehensive diagnostic panorama of major mental disorders among caregivers. Other codes were excluded due to their developmental origins or a low likelihood of being related to caregiving responsibilities.

Participants were classified as having major mental disorders if they had at least one visit to a specialized outpatient healthcare clinic or a hospital stay with an F-coded psychiatric diagnosis. In Finland, individuals with difficult-to-treat psychiatric symptoms and/or more severe mental disorders receive care at psychiatric outpatient clinics or in psychiatric departments. Those with mild psychiatric symptoms are typically treated by general practitioners within the primary healthcare settings and were, therefore, not included in our dataset or analysis here.

### Methods

We present continuous variables as mean and standard deviation or median and interquartile range. Categorical variables are summarized as frequencies and percentages of the total. Statistical significance was evaluated using two-way factorial models (analysis of variance or logistic regression models), where models included the main effects and the interaction effects. When analyzing the associations between caregiving status in 2012 and major mental disorders between 2012 and 2017, incidence rate ratios (IRRs) and 95% confidence intervals (95% CIs) for major mental disorders among caregivers compared with controls were calculated using generalized linear models using the log link and Poisson family (Poisson regression, follow-up time was exposure). Models were adjusted for age and SES.

A possible nonlinear relationship between the prevalence of major mental disorders and age was assessed using a three-knot-restricted generalized linear models using the log link and Poisson family (follow-up time was exposure). The length of the distribution of the knots was located at the 25th, 50th and 75th percentiles. The statistical analyses were conducted using the Stata 18.0 (StataCorp LP; College Station, Texas, USA) software package.

## Results

Table 1 presents the descriptive characteristics of caregivers and controls. The average age of the study population was 67 years (range 18–103). Overall, 5036 caregivers (11.9%) received at least one diagnosis of a mental disorder. The total number of F-coded diagnoses among caregivers was 6947. Among controls, 10,233 (12.2%) had at least one F-coded diagnosis, with a total of 13,515 F-coded diagnoses recorded. Controls without mental disorder diagnoses were the most highly educated group, while those with a mental disorder diagnosis had the lowest educational attainment. Across all groups, the majority were pensioners. Income and SES tended to be lower among individuals diagnosed with a mental disorder compared with those without such diagnoses. Caregivers diagnosed with a mental disorder had a higher SES than controls with similar diagnoses. Female caregivers had a higher educational attainment and were more likely to be employed or studying than male caregivers, whereas male caregivers had higher incomes and a larger proportion were retired (see supplementary material).

**Table 1.** Characteristics of Finnish informal caregivers and their matched (age, sex and municipality of residence) controls (total *n* = 125,874).

	Controls *n* = 83,618		Caregivers *n* = 42,256		*p* Values		
	Without F-code diagnosis	With F-code diagnosis	Without F-code diagnosis	With F-code diagnosis	Controls vs. caregivers	Without F-code diagnosis vs. with	Interaction
	*n* = 73,385	*n* = 10,233	*n* = 37,220	*n* = 5036	–	–	–
Women, *n* (%)	52,112 (71)	7029 (69)	26,341 (71)	3505 (70)	.44	<.001	.17
Age, in years (range)	66 (19–103)	70 (20–100)	67 (18–102)	66 (20–98)	<.001	<.001	<.001
Education, mean, years (SD)	12.2 (2.8)	11.4 (2.7)	11.7 (2.6)	11.6 (2.6)	<.001	<.001	<.001
Employment status, *n* (%)	–	–	–	–	<.001	<.001	<.001
Employed/student	25,137 (34)	1851 (18)	10,507 (28)	1236 (25)	–	–	–
Part-time worker	2603 (4)	574 (6)	1999 (5)	420 (8)	–	–	–
Pensioner	45,645 (62)	7808 (76)	24,714 (66)	3380 (67)	–	–	–
Income at baseline in €1000, median (IQR)	21.2 (13.5–32.5)	15.5 (11.1–23.7)	20.9 (15.1–30.4)	18.4 (13.8–25.8)	<.001	<.001	<.001
Socioeconomic status,^a^ mean (SD)	0.075 (0.839)	−0.266 (0.741)	0.008 (0.709)	−0.118 (0.671)	<.001	<.001	<.001

^a^ Measured as an average of rank-normalized years of education and total income.

SD: standard deviation; IQR: interquartile range.

**Figure 1.**
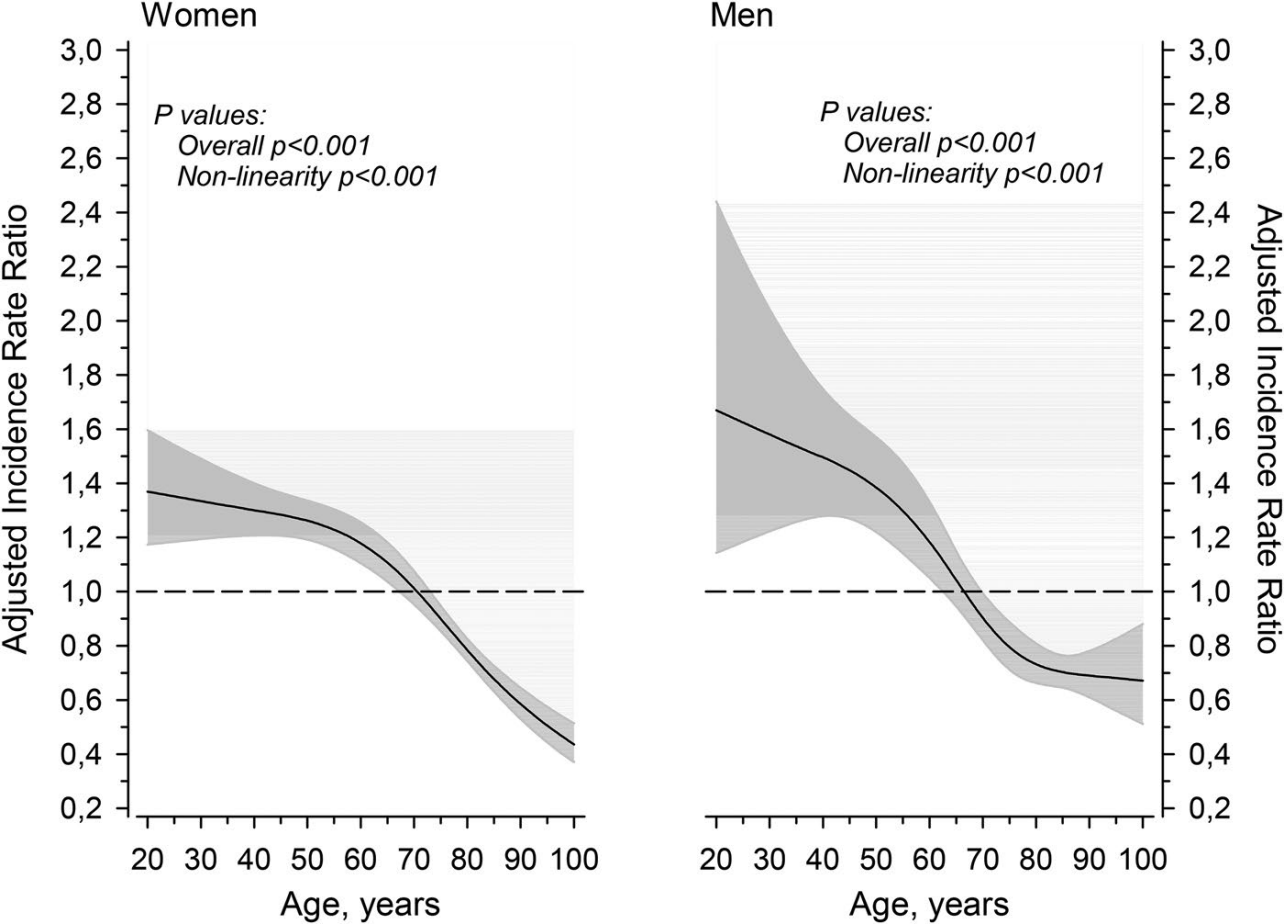
IRRs for a major mental disorder as a function of age. Curves were derived from three-knot restricted cubic spline generalized linear models. The models were adjusted for SES. The gray areas represent the 95% CIs.

IRRs of developing a major mental disorder among caregivers, accounting for age and sex, are illustrated in Figure 1. Both males and females exhibited the highest IRR of developing a major mental disorder in their early years (from age 20 onwards), surpassing that of controls, while the IRR was lower than that among controls at older ages (from age 70 onwards).

The most prevalent major mental disorders among caregivers were depressive and mood disorders (F32–F39) (Table 2). A diagnosis of depressive and mood disorders was found in 4.9% of caregivers vs. 3.6% of controls. The second most common major mental disorder among caregivers was anxiety-related disorders (F40–F45), affecting 3.7% of caregivers, vs. 2.6% of controls. The third most common major mental disorder among caregivers was mental disorders due to known physiological conditions, such as dementia (F00–F09). However, the diagnosis of dementia was present in 3.4% of caregivers vs. 5.4% of controls.

**Table 2.** Numbers of subjects with major mental disorder diagnoses and IRRs for ICD-10 F-code diagnoses among caregivers compared with controls.

			Incidence rate ratio (95% confidence interval)	
	Control *n* = 83,618 *n* (%)	Caregiver *n* = 42,256 *n* (%)	Crude	Adjusted for age and socioeconomic status
All, ICD-10 F-codes				
F00–F09	4526 (5.4)	1440 (3.4)	0.62 (0.58–0.65)	0.58 (0.55–0.61)
F10–F19	1717 (2.1)	844 (2.0)	0.95 (0.88–1.03)	0.93 (0.86–1.01)
F20–F29	1085 (1.3)	394 (0.9)	0.70 (0.63–0.79)	0.70 (0.62–0.78)
F30–F31	473 (0.6)	250 (0.6)	1.02 (0.88–1.19)	1.00 (0.86–1.17)
F32–F39	2992 (3.6)	2091 (4.9)	1.35 (1.28–1.43)	1.33 (1.26–1.41)
F40–F45	2202 (2.6)	1583 (3.7)	1.39 (1.31–1.48)	1.37 (1.29–1.46)
F50	82 (0.1)	38 (0.1)	0.90 (0.61–1.32)	0.87 (0.59–1.28)
F60–F61	378 (0.5)	236 (0.6)	1.21 (1.03–1.42)	1.16 (0.99–1.37)
F90	60 (0.1)	71 (0.2)	2.29 (1.62–3.23)	2.23 (1.58–3.13)
Total	10,233 (12.2)	5036 (11.9)	0.95 (0.92–0.98)	0.94 (0.91–0.97)
Women, ICD-10 F-codes				
F00–F09	2777 (4.7)	844 (2.8)	0.59 (0.55–0.64)	0.57 (0.52–0.61)
F10–F19	864 (1.5)	403 (1.4)	0.91 (0.81–1.02)	0.89 (0.79–1.00)
F20–F29	836 (1.4)	266 (0.9)	0.62 (0.54–0.71)	0.62 (0.54–0.71)
F30–F31	372 (0.6)	193 (0.6)	1.01 (0.85–1.20)	0.99 (0.83–1.18)
F32–F39	2453 (4.1)	1661 (5.6)	1.32 (1.24–1.40)	1.30 (1.22–1.38)
F40–F45	1834 (3.1)	1297 (4.3)	1.38 (1.28–1.48)	1.36 (1.27–1.46)
F50	77 (0.1)	36 (0.1)	0.91 (0.61–1.35)	0.88 (0.59–1.32)
F60–F61	320 (0.5)	187 (0.6)	1.14 (0.95–1.36)	1.10 (0.91–1.31)
F90	44 (0.1)	57 (0.2)	2.52 (1.70–3.74)	2.48 (1.67–3.66)
Total	7029 (11.9)	3505 (11.7)	0.97 (0.93–1.01)	0.96 (0.93–1.00)
Men, ICD-10 F-codes				
F00–F09	1749 (7.1)	596 (4.8)	0.65 (0.59–0.71)	0.61 (0.55–0.66)
F10–F19	853 (3.5)	441 (3.6)	0.99 (0.88–1.10)	0.96 (0.86–1.08)
F20–F29	249 (1.0)	128 (1.0)	0.98 (0.79–1.21)	0.96 (0.78–1.18)
F30–F31	101 (0.4)	57 (0.5)	1.08 (0.78–1.49)	1.08 (0.78–1.49)
F32–F39	539 (2.2)	430 (3.5)	1.52 (1.34–1.72)	1.49 (1.32–1.69)
F40–F45	368 (1.5)	286 (2.3)	1.48 (1.27–1.73)	1.45 (1.25–1.69)
F50	≤5	≤5	0.76 (0.15–3.93)	0.75 (0.14–3.95)
F60–F61	58 (0.2)	49 (0.4)	1.61 (1.10–2.35)	1.53 (1.04–2.23)
F90	16 (0.1)	14 (0.1)	1.67 (0.81–3.42)	1.57 (0.77–3.21)
Total	3204 (13.1)	1531 (12.3)	0.91 (0.86–0.96)	0.90 (0.85–0.96)

ICD-10: International Classification of Diseases, tenth revision; F00–F09: mental disorders caused by known physiological conditions, such as dementia; F10–F19: mental and behavioral disorders due to psychoactive substance use; F20–F29: schizophrenia, schizotypal, delusional and other non-mood psychotic disorders; F30–F31: manic episodes and bipolar disorder; F32–F39: depressive episodes, major depressive disorders, recurrent, persistent mood (affective) disorders and unspecified mood (affective) disorders (depressive and mood disorders); F40–F45: anxiety, dissociative, stress-related and somatoform mental disorders (anxiety-related disorders); F50: eating disorders; F60–F61: specific (adult) personality disorders; F90: hyperkinetic disorders, with ADHD being the most common subcategory; Total: any ICD-10 code F00–F90.

When comparing the likelihoods of major mental disorders in caregivers compared to controls using IRRs, caregiving was associated with a lower likelihood of major mental disorders, with an overall adjusted IRR of 0.94 (95% CI 0.91–0.97; Table 2). However, caregivers were more likely to experience depressive and mood disorders (F32–F39) with an IRR of 1.33 (95% CI 1.26–1.41), as well as anxiety-related disorders (F40–F45) with an IRR of 1.37 (95% CI 1.29–1.46), when compared with controls. The likelihood of these disorders was also higher among caregivers than in the controls when analyzing each sex separately. In addition, caregivers overall had an IRR for ADHD (F90) of 2.23 (95% CI 1.58–3.13), and 2.48 (95% CI 1.67–3.66) for female caregivers. Caregivers also had a higher likelihood of specific personality disorders (F60–F61) with a crude IRR of 1.21 (95% CI 1.03–1.42), but after adjustment this rate dropped to 1.16 (95% CI 0.99–1.37).

During follow-up, caregivers had a lower likelihood (IRR 0.58, 95% CI 0.55–0.61) of being diagnosed with major mental disorders caused by known physiological conditions, such as dementia (F00–F09), as well as non-affective psychotic disorders (F20–F29) with an IRR of 0.70 (95% CI 0.62–0.78) when compared with controls (Table 2).

**Figure 2.**
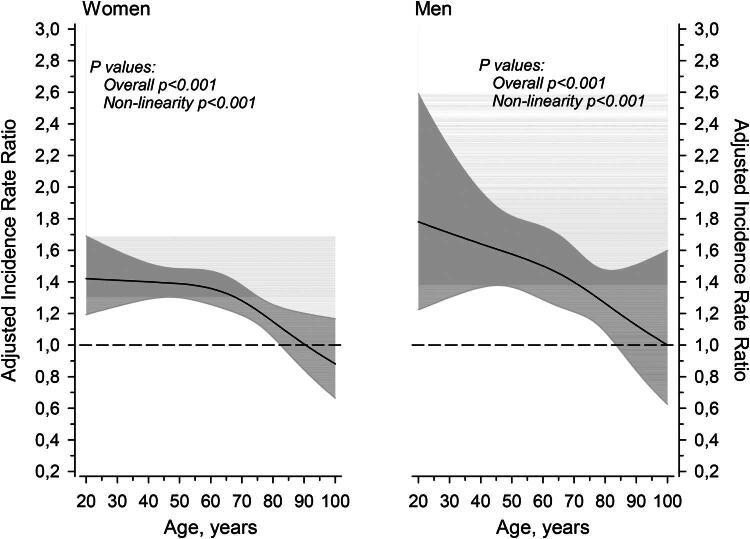
IRRs for having a depressive/mood disorder (F32–F39) or an anxiety-related disorder (F40–F45) as a function of age. Curves were derived from three-knot restricted cubic spline generalized linear models. The models were adjusted for SES. The gray areas represent the 95% CIs.

IRRs of having a depressive and mood disorder (F32–F39) or an anxiety-related disorder (F40–F45) as functions of age are illustrated in Figure 2. These subgroups were the two most common mental disorder subgroups in caregivers and were therefore examined separately. Among female caregivers aged 20–60 the IRR was about 1.4 times higher than in controls and remains nearly at the same level until age 60, after which the IRR gradually decreased with age and became comparable to that of controls around age 85. Among young male caregivers, the IRR was higher than among controls and gradually decreased with age, reaching the level of the controls by age 85. Compared to Figure 1, which illustrates the pattern across all F category diagnoses, these results indicate that the elevated likelihood of depressive and mood disorders (F32–F39) and anxiety-related disorders (F40–F45) persists longer than the likelihood observed for all F-diagnoses combined.

**Figure 3.**
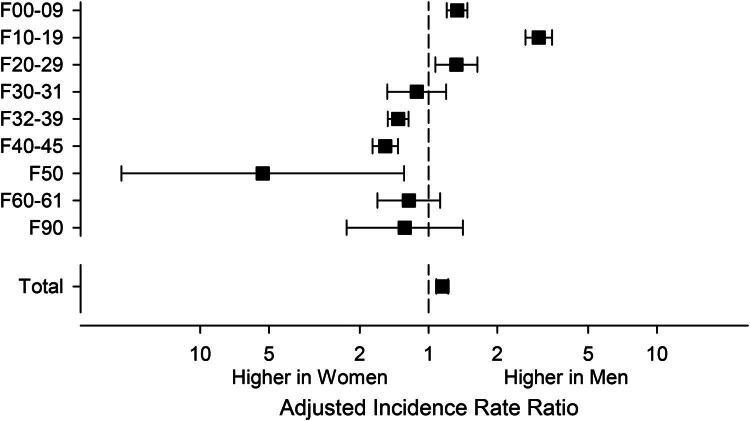
IRRs of major mental disorders diagnoses (ICD-10 F-codes) between female and male caregivers, adjusted for age and SES. ICD-10: International Classification of Diseases, tenth revision; F00–F09: mental disorders caused by known physiological conditions, such as dementia; F10–F19: mental and behavioral disorders due to psychoactive substance use; F20–F29, schizophrenia, schizotypal, delusional and other non-mood psychotic disorders; F30–F31: manic episodes and bipolar disorder; F32–F39: depressive episodes, major depressive disorders, recurrent, persistent mood (affective) disorders and unspecified mood (affective) disorders (depressive and mood disorders); F40–F45: anxiety, dissociative, stress-related and somatoform mental disorders (anxiety-related disorders); F50: eating disorders; F60–F61: specific (adult) personality disorders; F90: hyperkinetic disorders, with ADHD being the most common subcategory; Total: any ICD-10 code F00–F99.

When studying sex differences in IRRs among caregivers (Figure 3), we found that male caregivers had a higher probability of major mental disorders compared with female caregivers (IRR 1.15, 95% CI 1.08–1.22). Male caregivers also faced a greater probability (IRR 3.04, 95% CI 2.66–3.47) of mental and behavioral disorders related to psychoactive substance use (F10–F19) compared with female caregivers, as well as a higher likelihood (IRR 1.32, 95% CI 1.07–1.64) of non-affective psychotic disorders (F20–F29). By contrast, male caregivers had a lower probability (IRR 0.74, 95% CI 0.66–0.82) of depressive and mood disorders (F32–39), , anxiety-related disorders (F40–F45) (IRR 0.65, 95% CI 0.57–0.73) and eating disorders (F50) (IRR 0.19, 95% CI 0.05–0.78) compared with female caregivers.

## Discussion

Overall, our findings suggest that caregivers had a lower probability of major mental disorders compared to controls. Yet, the difference was small. However, younger caregivers faced a higher likelihood of a major mental disorder diagnosis, while older caregivers exhibited a lower likelihood for a major mental disorder compared with controls. In addition, male caregivers exhibited a slightly higher probability of major mental disorders than female caregivers. Caregivers were more likely than controls to experience several psychiatric conditions, including depressive and mood disorders, anxiety-related disorders, specific personality disorders and ADHD. Conversely, caregivers were less likely to experience major mental disorders caused by known physiological conditions, such as dementia and non-affective psychotic disorders, than controls.

Our findings indicate that caregiving is linked to a lower overall likelihood of major mental disorders. These findings support the healthy caregiver hypothesis, which suggests that adults in better health are more likely to become caregivers and remain in that role [32,33]. Nevertheless, in this study, the likelihood of experiencing a major mental disorder remained higher for caregivers than for controls until the age of 70. Subsequently, the likelihood dropped below that for controls. Interestingly, Doebler et al.’s [13] findings align with our observations, although the age ranges differed somewhat between their study and ours. Specifically, they reported an association between full-time caregiving and mental-ill health among caregivers under the age of 50, supported by both subjective mental health assessments and objective indicators, such as antidepressant prescriptions [13]. That said, their data sources differed substantially from our major mental health disorder data, which were derived from a national register covering all psychiatric diagnoses given in specialized healthcare settings or hospitals (not only depression). In contrast to our study, their analysis of individuals aged 50 and older showed that the association with full-time caregiving was minimal. Diverging from our results, a study by Grande et al. [34] which focused on end-of-life caregiving, reported higher levels of psychological morbidity among caregivers across all age groups compared with the general population. However, consistent with our findings on age-dependence, they observed that younger caregivers experience the highest rates of psychiatric morbidity, with rates decreasing as age increased. Supporting our findings, recent studies on young adult caregivers indicate that young caregivers experience worse mental health outcomes than their peers without caregiving responsibilities [35,36].

Evidence from several studies indicates that caregiving is associated with an increased likelihood of depressive and anxiety symptoms. The results of this study, which utilized data on diagnosed major depressive and mood disorders (F32–F39), as well as anxiety-related disorders (F40–F45) treated in a hospital or specialized care settings, align with previous studies examining depressive and anxiety symptoms [7–9]. In our study, caregivers were more likely than controls to have diagnoses of depressive and mood disorders as well as anxiety-related disorders. We also found that female caregivers were more likely than male caregivers to receive diagnoses of depressive and mood disorders and anxiety-related disorders. The sex distribution regarding the likelihood of these diseases in our study aligns with the results of previous meta-analyses [16,19], although severity levels varied significantly.

The burden associated with caregiving responsibilities is significantly correlated with increased symptoms of depression and anxiety in caregivers, with a higher burden predicting more severe symptoms. Specific factors thought to contribute to this heightened risk include persistent stress from caregiving duties, limitations on work and leisure activities, reduced social interactions and interpersonal conflicts arising from the caregiving role [4,9,37,38].

To our knowledge, the existing literature has not specifically addressed ADHD or specific personality disorders among caregivers. In our study, caregivers demonstrated a significantly higher likelihood of ADHD (F90) and higher likelihood, especially in men, of personality disorders (F60–F61) than controls. However, the relationship between caregiving and the development of these disorders remains inconclusive. Stress, life changes and comorbid conditions, such as anxiety and depression, may contribute to exacerbated ADHD symptoms [39,40]. Therefore, it is plausible that the elevated stress associated with caregiving may be associated with an increased probability of recurrence of these often-pre-existing disorders among caregivers.

Our findings also indicate that male caregivers were more likely than female caregivers to have non-affective psychotic disorders, such as schizophrenia (F20–F29), as well as disorders due to physiological conditions like dementia (F00–F09). Yet, these rates remained lower overall when compared with controls. Due to functional limitations, individuals with cognitive impairments or psychotic disorders may be unwilling or considered incapable of caregiving responsibilities.

Since most caregivers are women [41,42], male caregivers may go unnoticed in large research datasets, and their unique mental health challenges and disorders might be overlooked or underestimated. In our study, male caregivers had a higher likelihood than female caregivers of experiencing major mental disorders. Notably, we found no studies that specifically addressed gender differences in diagnosed mental disorders among caregivers. However, findings from a review by Yee and Schulz [43] indicated that female caregivers reported higher levels of psychiatric symptoms compared with their male counterparts.

The reasons for gender differences in morbidity rates may be influenced by gender-specific caregiving dynamics. Male caregivers tend to adopt a task-oriented approach, viewing caregiving as a job and seeking support when needed. By contrast, female caregivers often perceive caregiving as an emotional extension of familial roles, which can lead them to underestimate their own support needs [44]. That said, male caregivers might receive less social support than women, given that support systems are often designed to meet women’s needs and preferences. Men’s involvement in caregiving can promote social change and reshape gender roles [45]. However, the conflict between work identities and caregiving roles may cause stress for men until these standards change [46].

The key strengths of our study include its large sample size, the six-year follow-up period and the inclusion of all formally recognized family caregivers in Finland, along with a matched control group twice the size of cases. Furthermore, the diagnostic data were highly reliable, since all information was obtained from national administrative registers. The dataset also included demographic variables, such as age and sex, enabling analyses based on these characteristics.

When interpreting these research findings, it is important to remember that ∼80% of formally recognized family caregivers in Finland provide almost continuous and demanding care [27]. This indicates that our data primarily captures caregivers with a high caregiving burden and likely excludes those who provide lighter or intermittent care. Moreover, our data included psychiatric diagnoses recorded in hospitals and specialized healthcare settings, while excluding individuals who received treatment for mental health disorders in primary care contexts alone. As a result, the actual prevalence of mental disorders among family caregivers is likely higher, especially for milder and more common disorders, such as depression and anxiety. Additionally, it is likely that not all eligible individuals apply for the allowance, and those with severe mental illness may be somewhat underrepresented. Also, if caregivers differ from controls in how likely they are to seek healthcare, it might influence results, although the direction of this potential bias is unclear.

Furthermore, detailed information on the caregiver characteristics, care recipients (e.g. health status) and the nature of caregiving relationships – such as caregiving intensity and duration – are lacking. Also, our data on caregiver status and background variables are limited to the baseline year. These factors restrict the extent to which the underlying determinants of the association between caregiving and mental disorders can be examined and thereby limiting conclusions regarding causal relationships.

To understand causal relationships better, future studies could examine caregiving status and major mental disorders in a longitudinal setting with repeated information on both the exposure and outcome. Such analysis would also allow taking into account disorders prior to assuming the caregiver role and analyzing changes in mental morbidity even after ceasing the caregiver role. In the future, it would also be valuable to obtain research findings on whether caregivers and non-caregiving controls differ in their propensity to seek medical care, and whether patterns of health care usage vary between primary and specialized healthcare.

## Conclusions

This study sheds light on major mental health disorders among caregivers and their likelihood of developing specific mental disorders. Recognizing these probabilities fosters early support and preventive measures to protect caregivers’ mental health before problems arise. It also facilitates the early detection of potential disorders, ensuring that caregivers receive prompt help if they fall ill and that care recipients are provided with high-quality care.

## Supplementary Material

Supplementary material.docx

## Data Availability

The data that support the findings of this study are available from the Health Data Permit Authority Findata. Restrictions apply to the availability of these data, which were used under license for this study. Data are available from Findata/at Data - Findata with the permission of the principal investigator of Folkhälsan Research Center (Samfundet Folkhälsan i Svenska Finland rf) and Findata.
