# Supplementary material for: Major mental disorders among family caregivers: a nation-wide register-based study
Source: Scand J Prim Health Care. 2026 May 17;44(1):2671956. doi: 10.1080/02813432.2026.2671956 (PMC13182183; doi:10.1080/02813432.2026.2671956)
Supplement: Supplementary material.docx [file IPRI_A_2671956_SM7864.docx]

**Supplementary material**

Descriptive characteristics of Finnish informal caregivers (n=42 256) categorized by sex

|  | Women  n=29 846 | Men  n=12 410 | p-value |
| --- | --- | --- | --- |
| Age, in years (range) | 66 (18–103) | 71 (20–102) | <0.001 |
| Education, mean years (SD) | 11.8 (2.6) | 11.5 (2.6) | <0.001 |
| Employment status, n (%) |  |  | <0.001 |
| Employed/student | 9 447 (31.7) | 2 296 (18.5) |  |
| Part-time worker | 1 709 (5.7) | 710 (5.7) |  |
| Pensioner | 18 690 (62.6) | 9 404 (75.8) |  |
| Income at baseline in €1000, median (IQR) | 19.7 (14.5–28.8) | 22.8 (16.5–32.4) | <0.001 |
| Socioeconomic status, * mean (SD) | -0.02 (0.70) | 0.03 (0.71) | <0.001 |

*Measured as an average of rank-normalized years of education and total income.

Abbreviations: SD, standard deviation; IQR, interquartile range.
